# Factors influencing participation in breast cancer screening in an urban setting. A study of organized and individual opportunistic screening among potentially active and retired women in the city of Nice

**DOI:** 10.1016/j.pmedr.2022.102085

**Published:** 2022-12-06

**Authors:** Laurent Bailly, Thomas Jobert, Mirko Petrovic, Christian Pradier

**Affiliations:** a Université Côte d'Azur, Centre Hospitalier Universitaire de Nice, Department of Public Health, UR2CA, Nice, France; b Université Côte d'Azur, CNRS, GREDEG, 250 rue Albert Einstein, Valbonne 06560, France; c Université Côte d’Azur, EUR ELMI, Rue du 22ème B.C.A., Nice 06300, France

**Keywords:** Breast cancer screening, Adherence, Participation, Social determinants of health, Health disparities

## Abstract

Participation in breast cancer screening in urban settings is poor. Identifying factors accounting for participation and non-participation is essential to target priority areas, tackle health inequalities and suggest innovative approaches.

We studied organized and individual opportunistic participation in breast cancer screening within the 144 aggregated units for statistical information (Ilôts regroupés pour l’information statistique: IRIS) of the city of Nice from 2019 to 2021. In each IRIS was assessed a local human development index, among potentially active women aged 50 to 59 years and retired women aged 60 to 74 years. Modelling participation and non-participation in screening according to the IRIS units’ socio-economical characteristics was performed using the SURE method (Seemingly Unrelated Regression Equations).

Over a 2-year period, 24,396 breast screening tests were conducted (11,173 as organised screening, 13,223 as individual opportunistic screening). The local human development index was positively correlated with the two types of screening, respectively. Access to public transport facilitated participation. Managerial status was negatively correlated with organised screening. Single working women had a higher risk of non-participation. With regard to their socio-economic characteristics, screening rates were lower than expected in 16 IRIS units in the city of Nice.

Local human development index, access to public transport, family and professional context appear to be associated with breast cancer screening in an urban setting. An innovative approach targeting these factors is called for to reduce health inequalities.

## Highlights

• Over a 2-year period, 24,396 organized and opportunistic breast screening tests were conducted in an urban setting.
• The social gradient was positively correlated with the participation.
• Managerial status was negatively correlated with organised screening.
• Single working women had a higher risk of non-participation.
• Access to public transport facilitated participation.

## 1 Introduction

Breast cancer is the most frequent malignancy and the leading cause of death from cancer among women both in France (Bray et al., 2018) and in Europe (Dyba et al., 2021). Although they have been subject to controversy (Independent UK Panel on Breast Cancer Screening, 2012), universal breast screening programmes have been shown to reduce breast cancer mortality thanks to earlier diagnosis (Myers et al., 2015, Schünemann et al., 2020). For an organised breast cancer screening programme to be effective, participation of the target population should reach 70 % (Perry et al., 2008). In France, the participation rate for the organised breast screening programme was 48.6 % in 2018–2019 and 45.6 % in 2019–2020 (“Taux de participation au programme de dépistage organisé du cancer du sein 2019–2020 et évolution depuis 2005,” n.d.). However, these national participation rates reveal dissimilar situations when broken down to specific territories demonstrating the existence of social and territorial inequalities in France (Deborde et al., 2018), with low participation rates in both the least and the most affluent districts, as well as in urban centres and rural areas. Such differences in participation rates according to socio-economic characteristics and geographical areas have already been reported in England (Maheswaran et al., 2006) or in Sweden (Lagerlund et al., 2015), as well as in large cities such as Montreal (St-Jacques et al., 2013), Sydney (Khan et al., 2021) or London (Jack et al., 2016).

In urban areas offering an abundance of radiological facilities and gynaecologists, studies focusing on opportunistic breast cancer screening are scarce. However, European scientific societies have required opportunistic screening to be included among indicators monitoring screening campaigns (Muratov et al., 2020) thus allowing priority targeting of women not undergoing any breast cancer screening procedure. In France, opportunistic screening is usually prescribed as part of the gynaecological follow-up, particularly for women below the age required for organised screening, i.e. from 50 to 75 (“La participation au dépistage du cancer du sein des femmes de 50 à 74 ans en France,” n.d.). It can thus be more convenient for women already undergoing individual opportunistic screening (Heikkinen et al., 2016, Ouédraogo et al., 2014a) to continue doing so rather than to take part in organized screening programmes.

The aim of this study was to identify factors associated with participation or non-participation in breast cancer screening within an urban setting offering numerous medical facilities.

## 2 Method

### 2.1 Study population

Our study population consists of women aged 50 to 74 years with national health insurance coverage and living in Nice city, France. Screening data were extracted from the National Health Insurance medical department reimbursements database. Insured women not living at the recorded address and those recorded as deceased were excluded. Medical procedures were investigated on the basis of reimbursements for bilateral breast cancer screening by mammography according to codes contained in the Common Classification of Medical Procedures, i.e. QEQK001 for individual, opportunistic screening and QEQK004 for organized screening. Unilateral mammography, coded QEQK005, was excluded. To cover the 2-year interval recommended between each screening procedure, the study was conducted from June 17, 2019 to June 17, 2021.

There were 24,396 screening tests geo-located at the IRIS level (Merged Islet for Statistical Information) which is the smallest geographical unit in France (“Definition - IRIS | Insee,” n.d.) for which census data are available (Deborde et al., 2018), of which 11,173 resulted from organized screening and 13,223 from individual screening, out of an eligible population of 43,402 women. The total number of organized screening tests and eligible population were confirmed by the Regional Coordination Centre for cancer screening in the Provence Alpes Côte d’Azur region. The data related to the number of screenings is shown according to each IRIS unit. For the city of Nice, the total number of IRIS units is 144 and the participation rates we used for the study were grouped into two categories, i.e. women aged 50–59 and those aged 60–74 years, to differentiate between potentially working and retired women, since in France, beyond the age of 60, one woman out of two is retired (Minni, 2012). Those percentages were calculated for each type of screening and each age group, as the ratio of the number of women screened to the number of women in that age-group with national health insurance and living in the IRIS unit during this period.

IRIS unit social and economic characteristics were obtained from population census data collected by the National Institute for Statistics and Economic Studies (Insee) in 2018 and published in 2021. Variables relating to income, their origin and their distribution were obtained from the Localised Disposable Income System (Dispositif FiLosofi) data (“Dispositif sur les revenus localisés sociaux et fiscaux | Insee,” n.d.) collected in 2018 and published in 2021. The IRIS unit socio-economic characteristics are measured for the total population of each IRIS and not restricted to the eligible women. We assumed that Kelman’s identification concept could be applied to our age group of interest (Kelman, 1958): an individual changes his or her behaviour because he or she identifies with the individual or group that is the source of influence.

### 2.2 Ethical considerations

The study was approved by the Côte d’Azur University ethics board.

### 2.3 Local human development index (LHDI)

As an alternative to usual social deprivation indices, we chose to create a new, more suitable measure for this type of study to capture the level of development of an area. We will discuss the benefits of this indicator in the discussion section.

This index composite, dimensionless, between 0 (awful) and 1 (excellent) is based on work published by Mahbub ul Haq (Ul Haq, 1995) and Amartya Kumar Sen (Anand and Sen, 2000) and has since become the main metric used by the United Nations to quantify the “average achievement in key dimensions of human development“. However, instead of computing it at the national level, this was done at an IRIS unit level. The LHDI includes the same three components as the HDI: health, education, and income. The index is thus computed as follows:

$$\mathrm{LHDI}=\sqrt[{3}]{I_{\mathrm{health}}.I_{\mathrm{education}}.I_{\mathrm{income}}}$$

Hence, for the health index, we did not use life expectancy at birth since this is not available on a city-wide scale, but we used the share of male workers, considering the fact that, on average, their life is 6 years shorter than male executives (Blanpain, 2016). In the same way, for the education index, we chose to consider the share of the population without any degree rather than the mean years of schooling which is used at the international level. Finally, for the income index, we used the median disposable income.

The literature review suggests opposing contributions of the social gradient to explain participation in an organized breast cancer screening programme. On one hand, the social gradient may positively contribute to the organised screening rate (Deborde et al., 2018). On the other hand, considering that women on the upper end position on the social gradient regularly visit a gynaecologist, it can be expected that not all will take part in an organised screening campaign, and that some will favour individual screening prescribed by their own gynaecologist (Heikkinen et al., 2016). Belonging to the higher end of the social gradient may thus have a negative contribution to the organised screening rate (Rollet et al., 2021). We call this second contribution the “crowding out effect of individual opportunistic mammography screening (IMS) on organized mammography screening (OMS) » and assess it according to the percentage of women executives which can be negatively correlated with organised screening.

### 2.4 Statistical analysis

Compared to previous studies (Ouédraogo et al., 2014a, 2014b), we considered age group as the variable that should be explained by the model rather than used as an exogenous variable. Therefore, we had to solve 4 distinct equations, and to do so, we estimated them according to the Seemingly Unrelated Regressions (SUR) model (Zellner, 1962).

The SUR method is a generalization of the Ordinary Least Squares (OLS) model that involves specific equations, with distinct dependent variables. The errors are correlated across equations for a given individual but are uncorrelated across individuals. It allows testing the heterogeneity of behavior between groups, in our case between working and retired women, as well as for organized and opportunistic mammography screening.

The model is constructed as follows:

$$y_{\mathrm{ij}}=x_{\mathrm{ij}}^{\prime }\beta +u_{\mathrm{ij}}$$

The model consists of j = 4 linear regression equations for i = 144 IRIS.

We can stack the m equations into a SUR model:

$$\left[\begin{array}{c} y_{1}\\ y_{2}\\ y_{3}\\ y_{4} \end{array}\right]=\left[\begin{array}{ccc} X_{1} & 0\cdots & 0\\ \vdots & \ddots & \vdots \\ 0 & \cdots & X_{4} \end{array}\right].\left[\begin{array}{c} \beta_{1}\\ \beta_{2}\\ \beta_{3}\\ \beta_{4} \end{array}\right]+\left[\begin{array}{c} u_{1}\\ u_{2}\\ u_{3}\\ u_{4} \end{array}\right]=X\beta +u$$

With:

$$\left\{\begin{array}{c} \begin{array}{c} {\begin{array}{c} y \end{array}}_{1}=Percentage\;of\;organized\;mammography\;screenings\;for\;working\;age\;women\\ y_{2}=Percentage\;of\;organized\;mammography\;screenings\;for\;retired\;women\\ y_{3}=Percentage\;of\;opportunistic\;mammography\;screenings\;for\;working\;age\;women \end{array}\\ y_{4}=Percentage\;of\;opportunistic\;mammography\;screenings\;for\;retired\;women \end{array}\right)$$

OLS estimation for each equation yields a consistent estimator of β, but the optimal estimator is the generalized least squares (GLS) estimator:

$${\hat{\beta }}_{\mathrm{GLS}}={\left\{X^{\prime }\left(\Sigma^{-1}⊗I_{N}\right)X\right\}}^{-1}\left\{X^{\prime }\left(\Sigma^{-1}⊗I_{N}\right)y\right\}$$

where ⊗ is the tensor product and $I_{N}$ the identity matrix.

In addition to the LHDI, we implemented other explanatory variables in our model that describe the characteristics of the population of the IRIS unit, for instance the type of household, or the use of public transport. The variables were chosen using forward stepwise selection and were also based on previous literature pertaining to the subject of breast cancer. Thus, the variables we identified are the percentage of women executives, the percentage of the population using public transport to reach the workplace, the percentage of households without a car and the percentage of single persons aged between 55 and 79 years.

We will perform the homogeneity test later, to test whether the 4 equations are significantly different.

## 3 Results

Table 1 shows the summary statistics of the variables used in this study. Comparing screening rates, in the working age group (50–59 years), the percentage of IMS is on average significantly higher than the percentage of OMS. On the other hand, OMS and IMS are similar on average for the retired women group.

**Table 1:** Characteristics (Minimum, Maximum, Mean, Standard deviation) of 144 Nice IRIS’s in term of organized mammography screening (OMS), individual mammography screening (IMS), and non-participation screening, between 2019 and 2021.

Variable (effectives for city of Nice)	Minimum IRIS (IRIS effectives)	Maximum	Mean	Standard deviation
OMS rate 50–59 years old (3830 women)	8 % (4)	37 % (67)	21 % (27)	5,2%
OMS rate 60–74 years old (7343 women)	17 % (12)	41 % (104)	28 % (51)	7,3%
IMS rate 50–59 years old (5936 women)	15 % (14)	52 % (99)	33 % (41)	7,6%
IMS rate 60–74 years old (7287 women)	11 % (8)	46 % (129)	28 % (51)	7,2%
non-participation rate 50–59 years old (8 154 women)	23 % (19)	67 % (134)	46 % (57)	8,9%
non-participation rate 60–74 years old (10 942 women)	25 % (33)	66 % (135)	44 % (76)	8,5%

The regression tables derived from the SUR model (Table 2) suggest that the selected variables have a bigger impact on the OMS participation rate than on the IMS one.

**Table 2:** Regression table of the SUR model.

Variable / Equation	OMS 50–59		OMS 60–75		IMS 50–59		IMS 60–75	
	Coefficient	P-Value	Coefficient	P-Value	Coefficient	P-Value	Coefficient	P-Value
**Constant**	**0,146**	0,052	**0,188**	0,010	**0,152**	0,060	−0,066	−0,322
**Women executives**	**−0,747**	0,034	**−0,793**	0,020	0,489	0,194	0,123	0,692
**LHDI**	**0,168**	0,042	**0,158**	0,047	**0,318**	< 0,01	**0,485**	< 0,01
**Public transport**	**0,277**	< 0,01	**0,252**	< 0,01	0,045	0,480	0,046	0,389
**Without a car**	**−0,191**	< 0,01	**−0,195**	< 0,01	**−0,166**	< 0,01	**−0,084**	0,025
**Single women**	**−0,097**	0,084	0,012	0,821	−0,087	0,148	−0,023	0,644

First, there is a crowding-out effect of the OMS by the IMS, i.e. the share of women executives has a negative impact on OMS with an elasticity equal to −0.7 % while the impact is not significant for IMS.

As regards the LHDI, the human development index has a bigger impact on IMS than on OMS. The use of public transport has a positive impact on both organized screenings and age group whereas it is not significant for the IMS.

Not having a car is a barrier to testing, no matter the type of mammography or the age group. Finally, the share of single persons aged between 55 and 79 years has a negative effect on organized screening in the active women equation.

To evaluate the robustness of our study, the regression model explaining the non-participation rate was also examined. This concerned a larger population per IRIS unit since we did not separate IMS from OMS. This method should validate the choice of exogenous variables in the screening model as well as the hypothesis of a crowding-out effect where the share of women executives should not explain the non-participation rate. It reveals the differences between working and retired women. Finally, it shows the areas which under-perform or over-perform conditionally to their socio-economic characteristics (Table 3).

**Table 3:** Regression table of the non-participation rate for working and retired woman.

Variable /Equation	50–59		60–75	
	Coefficient	P-Value	Coefficient	P-Value
**Constant**	**0,701**	< 0,01	**0,877**	< 0,01
**Women executives**	0,258	0,549	0,670	0,082
**LHDI**	**−0,486**	< 0,01	**−0,644**	< 0,01
**Public transport**	**−0,323**	< 0,01	**−0,298**	< 0,01
**Without a car**	**0,357**	< 0,01	**0,279**	< 0,01
**Single women**	**0,184**	< 0,01	0,010	0,86

The share of women executives does not appear as a significant variable both with Student's *t*-test and Fisher's exact test (P-value = 0.069) which confirms the hypothesis of a crowding-out effect. The human development index has a positive impact on the general participation rate, especially for retired women. The effect of public transport is negative for both age groups which means that the easier it is to access public transport, the lower the non-participation rate. The opposite effect is observed for the share of people without a car, where a higher share is associated with a lower participation rate, this effect being stronger for working women.

We chose to present the results of the unconstrained model that allows for heterogeneity in screening behavior, both in terms of type of screening (OMS/IMS) and type of population (Active/Retired). As explained above, the SUR model allows us to test the heterogeneity of behavior between groups, so we needed to test whether our equations were significantly different. To do so, we developed a table with different constraints and a comparison of the F-tests showed whether behavior was homogenous or not between the groups, (i.e. OMS/IMS and working/retired woman). Table 4 shows that the hypothesis of homogeneity should be rejected since behavior is heterogeneous between groups. Table 5 confirms the same assumption as the Table 4, but this time for the non-participation rate model.

**Table 4:** Homogeneity test for SUR model.

Constraints	Number of constraints	Interpretation	F value	P. Value
$\beta_{1}=\beta_{2}=\beta_{3}=\beta_{4}$	18	Assumption of total homogeneity	40,63	<0,01
$\beta_{1}=\beta_{2}\beta_{3}=\beta_{4}$	12	Assumption of homogeneity between working and retired woman	22,09	<0,01
$\beta_{1}=\beta_{3}\beta_{2}=\beta_{4}$	12	Assumption of homogeneity between OMS and IMS	60,07	<0,01
$\beta_{1}=\beta_{2}$	6	Assumption of homogeneity between working and retired woman within the OMS	30,78	<0,01
$\beta_{3}=\beta_{4}$	6	Assumption of homogeneity between working and retired woman within the IMS	14,63	<0,01

**Table 5:** Homogeneity test for the non-participation rate model.

Constraints	Number of constraints	Interpretation	F value	P-value
$\beta_{1}=\beta_{2}$	6	Assumption of homogeneity between working and retired woman	3,44	<0,01

The performance level for participation in breast cancer screening in each IRIS unit in the city of Nice, given the characteristics included in the preceding models, is shown on map A. Among the 144 IRIS units, 16 were identified as having the lowest screening attendance rate. The deciles for the Human Development Index according to IRIS units are shown on map B. Comparing these two maps reveals that only 5 IRIS units among the 16 with the lowest performance rates are also those on the lowest level of human development index, i.e. the lowest decile of the HDI. In six of the 16 IRIS units with the lowest performance rates for each type of breast cancer screening, the HDI was either median or above the mean.

## 4 Discussion

Our study, which was conducted in an urban setting, showed that human development index, access to public transport, family context and professional status were key factors in breast cancer screening uptake, whether organised or opportunistic. A major finding is the proportion of individual screening among the breast cancer screening opportunities in a city with a high level of medical care facilities. Between June 2019 and June 2021, individual screening was far more frequent than organised screening, while at the national level it is estimated to account for only 10 % of breast cancer screening uptake in France (SPF, n.d.).

Modelling the rate of breast cancer screening confirmed the major role of the human development index, in screening participation, whether organised or individual. The effect of human development level was greater for individual screening than for organized screening, suggesting the former is directly linked with a high income: this confirms results obtained in France following studies focusing on individual data (Duport et al., 2008). Similarly, the proportion of women executives was negatively correlated with organised screening but was not statistically significant for individual screening. The link between level of education and more frequent individual screening has already been shown (Heikkinen et al., 2016). The socio-economic status of these women appears to result in avoidance of public prevention campaigns.

Instead of using usual social deprivation indices, we have developed a local human development index (LHDI). We have seen previously that the majority of studies have individual data. In this context, the Fdep can be used to construct a qualitative variable (the social gradient) as an explanatory variable in a (multinomial) logistic regression. Since we are working on quantitative data at the IRIS level with linear regression models, we cannot use qualitative variables. Rather than using usual deprivation indices we thought it would be appropriate to establish a new index for the reason that we will articulate in this segment. However, the discussion between the use of Fdep and LHDI should be seen as a purely statistical and academic discussion. In reality, when we compare the two indicators (LHDI and Fdep), they are highly correlated for the city of Nice with a linear negative correlation coefficient equal to 0,97. If we take the Fdep, as currently the referent health index, we can see that it was developed from a principal component analysis (PCA) of variables (Rey et al., 2009). That causes the problem of endogenous weighting because the result of the PCA is used as an explanatory variable. Because of that, there is also the problem of stability across time and space since the weights are changing as the sample changes. Finally, the fact that the index has an employment component introduces instability because the screening data we have are from the 2019–2021 period, which corresponds to the time when employment was largely disrupted by the effects of the COVID-19 pandemic, so a major bias would be introduced by using this index.

Our study also shows the role of the built environment on participation in prevention programmes: access to public transport is positively associated with organised breast cancer screening. Women who do not have access to public transport participate less in breast cancer prevention campaigns. The impact of transport facilities on organised screening programmes has already been demonstrated in 13 of the 95 administrative districts (départements) of metropolitan France (Ouédraogo et al., 2014b), revealing that having to travel of more than 15 min limited participation. In an urban setting, such a delay can easily be extended according to the place of residence, especially in a city such as Nice that covers many steep hills. Some areas in the city are easier to reach by car than by public transport, which may also explain the negative association between the proportion of households without a car and participation in screening, particularly organised screening. Accessibility (Linsell et al., 2010) of breast cancer screening facilities is of particularly importance and constitutes a major challenge in urban settings to reduce health inequality (Zidar et al., 2015).

Modelling non-attendance of screening, based on the variables included in the model that was developed to compute the screening rate in order to confirm its robustness, showed the role of family circumstances. There was less participation when the proportion of working women living alone was higher. Lower attendance of screening programmes by single, divorced or separated women has already been reported in French-speaking Swiss cantons (Bulliard et al., 2004).

Our model for non-participation in breast screening also revealed areas where screening was poor due to residents’ socio-economic, family or professional characteristics as well as access to public transport. It was thus possible to map both the human development index and the expected participation in those residential areas. A comparison of these two maps shows that deprived neighbourhoods are not the only ones to exhibit poor screening participation, while it was previously understood that individual screening in affluent urban areas compensated lower participation in organised screening. In France, such lower participation in non-deprived areas has already been shown in urban settings, such as in the city of Lyon (Padilla et al., 2019). In that study, fewer women living in the city centre underwent breast cancer screening compared to other city-dwelling women. This information is of paramount importance to guide public health policies targeting these areas of low participation rates which had not been previously identified.

A limitation of our study is the fact that we worked on aggregated and not on individual data, with no possibility to adjust on the age group for cancer screening (Walker and Becker, 2005). However, it has been shown that aggregated data could provide a reasonable approximation of social health inequalities (Pampalon et al., 2009). Moreover, several French studies using individual data revealed the same association between socio-economic level and participation in breast cancer screening (Duport et al., 2008, Menvielle et al., 2018, Menvielle et al., 2014). Our assumption that health-related attitudes and habits may be similar within IRIS units which would imply that residents of a particular area identify with the neighbourhood and conform their behaviour to that of their personal environment has already been demonstrated by others (Ouédraogo et al., 2014b). Another limitation relates to the study period which coincided with the COVID-19 pandemic which swept through France from March 2020 with a country-wide lockdown and a time when investigations not considered as urgent were postponed. In the city of Nice, screening tests declined during the lockdown, similarly for organized and opportunistic screening. Factors related to breast cancer screening practices could be different outside the context of a pandemic. An analysis of participation rates in organised screening at the local, regional, and national level (“Taux de participation au programme de dépistage organisé du cancer du sein 2019–2020 et évolution depuis 2005,” n.d.) suggests however that most screening tests were nevertheless undergone, since the difference with the previous campaign was only of 3 %. Lastly, the use of mammography reimbursement codes has not been evaluated for distinguishing between organised and individual screening and the CCAM code for bilateral mammography, used in the case of individual screening, does not distinguish between symptomatic and asymptomatic cases. However, since codes for unilateral mammography were excluded from the study, the percentage of symptomatic patients among the total number of tests performed should thus be limited.

### 4.1 Conclusion

Our study identified factors accounting for participation in breast cancer screening in an urban setting based on census and fiscal data: the human development index, access to public transport, the proportion of households possessing a car and the proportion of working women executives or those living on their own. Applying these criteria led to identification of 16 IRIS units where participation was below the expected rate. Searching for determinants of participation at the local level allows to adapt prevention efforts to the specific context of each urban area. This is an essential step if one wishes to target priority populations and to suggest innovative prevention measures to effectively reduce health inequalities. Qualitative studies should now be conducted involving representatives of the various geographical areas identified as having insufficient participation rates in breast cancer screening. Possible innovations, such as planning suitable screening times or creating dedicated transport facilities should first be discussed with representatives of the populations involved and tailored to the context of each territory.

## Funding

The Nice Côte d’Azur Metropolis funded the research involved for the Syndemie project.

## CRediT authorship contribution statement

**Laurent Bailly:** Conceptualization, Data curation, Formal analysis, Investigation, Methodology, Writing – original draft, Writing – review & editing. **Thomas Jobert:** Conceptualization, Data curation, Formal analysis, Investigation, Methodology, Writing – original draft, Writing – review & editing. **Mirko Petrovic:** Conceptualization, Data curation, Formal analysis, Investigation, Methodology, Writing – original draft, Writing – review & editing. **Christian Pradier:** Conceptualization, Data curation, Formal analysis, Investigation, Methodology, Writing – review & editing.

## Declaration of Competing Interest

The authors declare the following financial interests/personal relationships which may be considered as potential competing interests: Laurent Bailly is a member of the Regional Coordination Centre for cancer screening in the Provence Alpes Côte d’Azur region.

## Figures and Tables

**Supplementary figure 1:**
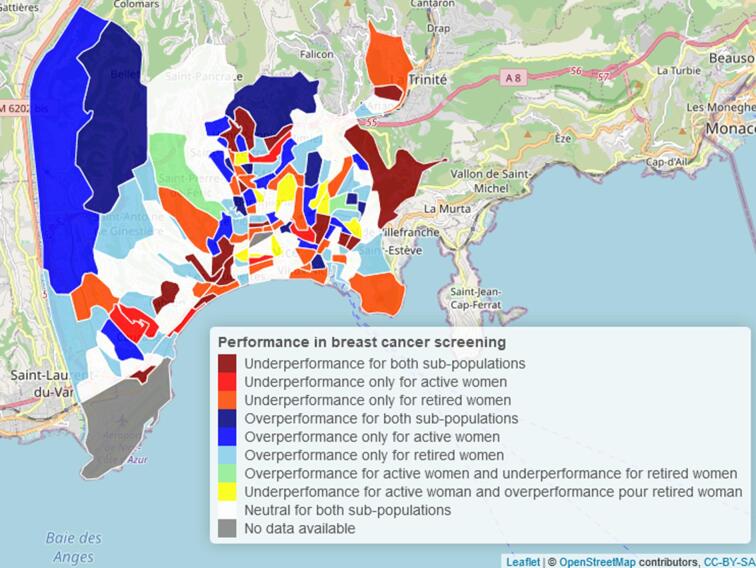


**Supplementary figure 2:**
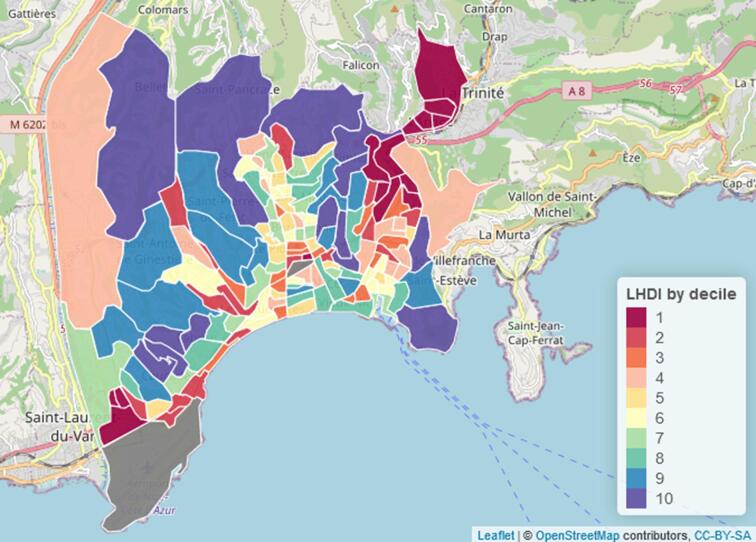


## Data Availability

The authors do not have permission to share data.
